# The challenges of ethical behaviors for drug supply in pharmacies in Iran by a principle-based approach

**DOI:** 10.1186/s12910-020-00531-0

**Published:** 2020-09-01

**Authors:** Mahla Iranmanesh, Vahid Yazdi-Feyzabadi, Mohammad Hossein Mehrolhassani

**Affiliations:** 1 Health Services Management Research Center, Institute for Futures Studies in Health, Kerman University of Medical Sciences, Haft-Bagh Highway- Kerman University of Medical Sciences, Kerman, Iran; 2 Social Determinants of Health Research Center, Institute for Futures Studies in Health, Kerman University of Medical Sciences, Kerman, Iran

**Keywords:** Ethical behaviors, Deontological approach, Drug supply, Pharmacy, Dignity and autonomy, Beneficence, Non-maleficence, Justice

## Abstract

**Background:**

Pharmacists as the trustee of pharmacy services must adhere to ethical principles and evaluate their professionalism. Pharmacists may sometimes show different unethical behaviors in their interactions, so it is essential to understand these behaviors. The present study aimed to determine the challenges of ethical behaviors based on a principles-based approach in the area of drug supply in pharmacies.

**Methods:**

This qualitative content analysis was conducted in Kerman in 2018. A number of key players in the field of medication supply were selected using snowball sampling to interview. An effort was made to select samples with maximum variation. Exclusion criteria include having less than 3 years of work experience in pharmacy and supervision, not willing to participate in the interview, and not participating in the interview for 3 times. The participants in this study consisted of pharmacy technicians (*n* = 5), patients (*n* = 6), pharmacists (*n* = 8), inspectors of insurance companies (*n* = 4), and inspectors of food and drug administration (*n* = 3). Data were analyzed using directed content analysis by Maxqda software version 10 (VERBI Software, Berlin, Germany). The principles of “Beauchamp and Childress Ethics” theory including autonomy, beneficence, non-maleficence, and justice were selected as the main principles.

**Results:**

After data analysis, 8 main categories and 26 subcategories were obtained. The main categories include patient privacy, patient independence, communication principles, patient-centered services, drug supplier, patient harm avoidance, supervision, and distributive, procedural, and interactional justice. The subcategories include increasing patient awareness, culturizing prescription, and rational drug use, confidentiality and privacy, and pharmacist-patient relationship/communication, which were the main ethical challenges in the area of drug supply at pharmacies.

**Conclusions:**

According to a principle-based approach, the greatest challenges were related to two principles of autonomy and beneficence. The policymakers in the healthcare system should emphasize patient independence, patient privacy, and patient-centered services. The results of this study can be used as a tool to introduce ethical challenges to policymakers and develop educational contents, the chart of professional ethics in pharmacies, and accreditation measures of pharmacies.

## Background

The function of the health system is in many ways dependent on human resources and is influenced by their behavior and ethics. Today, due to technological advances and complexity of human behavior, application of ethics in various professions is of great importance. Service providers tend to unnecessarily increase the use of healthcare services to gain profits, and patients have no choice but to purchase the service because of lack of information [1].

Among professional occupations, pharmacy as a profession with commercial and therapeutic dimensions [2, 3], has some specific complications [4]. Thus, pharmacists or pharmaceutical technicians are exposed to financial incentives [5] and may not be able to withstand against them [6]. Hoarding, black market, inter-professional trading, and unclear pharmaceutical policies are some examples of ethical dilemmas that pharmacists or pharmaceutical technicians are faced with [7, 8] and endanger consumers’ health.

The ethical considerations of pharmacists in the delivery of pharmaceutical care are influenced by various factors including physicians’ decisions, rules and regulations, guidelines, and marketing strategies for pharmaceutical industry. Also, ethics is the result of interaction between commercialism, consumerism, and pharmaceutical services [9]. Therefore, pharmacists as providers of pharmaceutical services, in any role and position, must adhere to ethics and evaluate their professional performance based on the ethical principles [10].

Since ethical considerations make sense within the context of ethical system, five ethical systems have been developed as the dominant systems at different times in the world [8], including 1) utilitarian system, 2) deontological system, 3) justice system, 4) libertarian system, and 5) benevolent system. It should be noted that there are other ethical systems based on the school of thoughts and religions that have proposed different definitions for specific words, such as justice and benevolence, which can be interpreted differently in different discourses [10, 11]. Ethical principles also vary in different ethical systems. Thus, as a considerable proportion of theories in the medical ethics philosophy is based on the principle of duty, the deontological system has been used to explain and justify ethical studies.

In a qualitative study by Cooper et al. (2007), on ethical problems and solutions among British pharmacists, two approaches were finally investigated for solving ethical problem. Kant’s deontological approach is one of the most controversial theories in the realm of moral philosophy, which proposes three principles including generalizability, respect to the dignity of individuals, and lawmaking for the moral community. The theory of Beauchamp and Childress is consisted of 4 principles, including autonomy, beneficence, non-maleficence, and justice, which are the basis for ethical decision-making in medicine. He also proposed a model of ethical decision-making in the areas of business and healthcare ethics [12].

The deontological approach of Ross (2012) also outlined duties including loyalty, gratitude, charity, justice, self-improvement, and non-maleficence. He also stated that some tasks are more imperative than others and can help physicians and pharmacists solve their ethical problems [13].

There is insufficient evidence to address the challenges of ethical behavior in the area of drug supply in pharmacies. Many international studies have used a questionnaire containing ethical scenarios and respondents had to select an option [14]. Some studies analyzed the frameworks for pharmaceutical ethics [12]. Kruijtbosch et al. (2017) highlighted the awareness and insight into the ethical aspects of working in pharmacies and found 9 steps for analyzing ethical dilemmas and solving problems [15]. However, many of these examples used in various studies are related to American, British, Swedish, and Australian pharmacists [12, 16]. Lowenthal et al. (1986) conducted a study in the United States to examine the attitudes of pharmacy students towards ethical dilemmas and compared them with those of the pharmacists and found that the main problem of pharmacy students was economic issues, and ethical issues should be discussed in pharmacy curriculum [17]. In Iran, in order to support and conduct the work process and decision-making by those who are working in the pharmaceutical profession, a comprehensive document of ethics in the pharmaceutical system was approved and announced by the Policy Council of the Ministry of Health in 2012 [18]. In 2015, a study examined the attitudes and experiences of pharmacists and faculty members of Shiraz School of Pharmacy towards ethical challenges in pharmacies [19]. In another study by Rasam (2008), the importance of paying attention to ethics pathology in drug therapy process was emphasized [7].

Many previous studies have mainly focused on the investigation of ethical issues quantitatively and no qualitative study has been found on the ethical issues in pharmacy settings using a four-principles-based approach. Thus, the present study was conducted to examine the perspectives and experiences of a wide range of stakeholders involved in pharmacy settings about ethical behaviors that occurred in the area of drug delivery systems using four principles of Beauchamp and Childress including autonomy, beneficence, non-maleficence, and justice. The results of the present study are expected to provide some advises about adopting tailored policies to improve ethical behaviors by identifying ethical challenges in the area of drug supply in pharmacy.

## Methods

### Study design

This qualitative study was conducted using the content analysis approach in Kerman, southeastern Iran, in 2018. Interviewees were selected from key players in the field of drug supply using snowball sampling method with maximum variation. Using this method, those who had a deep understanding of the ethical behaviors in pharmacy settings were included. Exclusion criteria were having less than 3 years of working experience in pharmacy and supervision, not willing to participate in the interview, and not participating in the interview for three times. The participants were recruited from pharmacy technicians (*n* = 5), patients (*n* = 6), pharmacists (*n* = 8), inspectors of insurance companies (*n* = 4), and inspectors of food and drug administration (*n* = 3). Demographic characteristics of the participants are shown in Table 1. The interviews continued until no new information was emerged from the interviews. To ensure data saturation, two additional interviews were conducted.

**Table 1:** General characteristics of the interviewees

Interview environment	Field of activity	Number
drugstore	Pharmacist	8
	Technician	5
	Patient	6
Insurance	Insurance Inspector	4
Food and drug administration	Food and drug administration inspector	3
Interviewees		23

### Data collection

Semi-structured in-depth interviews were performed between June and October 2018. To ensure that all relevant challenges would be identified, a topic guide was developed consisted of a four-principle-based approach. The approach, which is known as the principlism approach to biomedical ethics, is proposed by Beauchamp and Childress. It is one of the most important methodological inventions of modern practical ethics. The moral principles covered by this approach include autonomy, beneficence, non-maleficence, and justice. In this approach, the moral principles determine a particular moral situation [20]. To verify and order the number of questions, the primary interview guide was piloted on two non-participants, and the interview guide with few changes in the number of questions, ordering, and probe questions were finalized (See Supplementary 1). Initially, the interviewees were contacted in-person or via phone or email, and informed about the study topic, its objectives, and reasons for doing the present study. If they accepted the interview meeting, interviews were conducted at the interviewee’s office or any places agreed upon by the participants. Interviews were conducted by the first author of the study (MI) with a research background in the pharmaceutical sector, using the interview guide. All interviews were audio-recorded and immediately transcribed to be used as a guide in the subsequent interviews. Interviews lasted 30–55 min with an average time of 40 min. The interviewer also took field notes to share with the research team to discuss the initial findings, complications, and any modifications needed in the interview guide. Interviews were continued until data saturation. After data saturation when no additional data was emerged, two new interviews were also conducted. In total, 26 face-to-face semi-structured interviews were performed.

### Data analysis

Directed content analysis approach with a deductive coding was used to analyze the transcribed documents and categories development. The purpose of this approach is to validate or develop a theoretical framework or predetermined categories [21]. The four-principle approach for biomedical ethics, proposed by Beauchamp and Childress, was chosen as the main principle to categorize the data. The sub-principles were also extracted from the interviews and tailored to the relevant core principles. Each transcribed interview was read several times to localize the sections of transcribed interviews to deductive categories of the theory. The directed content analysis was conducted using the following steps. First, two of the authors (MI and MHM) independently coded the data and immersed themselves in data by reading and re-reading the transcribed documents, as well as listening to the recorded interviews. Then, the key principles of the approach were identified as the initial coding categories. Afterwards, operational definitions for each category were provided using the four-principle approach. Finally, any other text that could not be categorized with the initial coding scheme was given a new code. To reach an agreement on the relationship between the theory and text sections, all the authors held frequent sessions to discuss disagreements. The sessions were continued until all of the disagreements were addressed. In this step, categories and sub-categories were identified. At the end, the statements coded were reviewed, refined, and collated, and the results were finalized. The transcribed documents were analyzed using Maxqda version 10 (VERBI Software, Berlin, Germany).

### Trustworthiness of results

To enhance the trustworthiness of the results, Lincoln and Guba’s (1985) trustworthiness criteria [22] including credibility, dependability, confirmability, and transferability, were used. To ensure the credibility of the findings, a maximum variation sampling method was used to obtain diversity in interview participants to reveal multiple perspectives about the challenges of ethical or moral behaviors in pharmacy settings. It also helps improve the representativeness by seeking a wide range of ethical challenges in pharmacies. To enhance the credibility of the results, the interviews were conducted and analyzed over a seven-month period, which led to the prolonged engagement of the research team to immerse in the data, as well as allocating sufficient time for data collection, good communication with research participants, and taking notes and memos during interviews. Also, member checking technique was used to clear ambiguities of some interviews by which the coded documents returned to some interviewees to ensure that the codes obtained from the interviews were properly understood and the required modifications were made based on their comments. Moreover, after coding the transcribed documents by the two members of the research team independently, the codes were also reviewed in the presence of the third research member (VYF) in frequent sessions and the inconsistencies were discussed until a consensus was reached.

To ensure the dependability of the study, an audit approach, in which the authors accompanied with external auditors engaged in the complimentary comments, was recruited to cross-check, investigate inconsistencies, and address them to achieve a consensus. To improve confirmability, a research team with different research backgrounds, which can foster dialogue, conducted the present study, and subsequently, helps develop complementary, as well as divergent understandings of a study situation. It also allows that their values or theoretical inclinations to conduct the research did not affect the conducting the study. To ensure the transferability of the results, a series of quotations were embedded in the text as well as thick descriptions of all stages of the study, and the study setting was described, so that the reproducibility of the study was enhanced. Furthermore, the data were collected and analyzed at the same time.

### Ethical considerations

All participants voluntarily participated in this study. A study information sheet containing sufficient details about the present study was given to all participants before setting the interview schedule. They were assured that the anonymity and confidentiality of any information would be maintained. In order to record audio digitally, informed consent was verbally obtained from all participants before conducting the interviews. The present study was approved by the Ethics Committee of Kerman University of Medical Sciences (Ethical code: IR.KMU.REC.1397.567). No personal information was reported in this study or presented in any publications arising from the study. All names are position-based (Pharmacist, Technician, Patient, Insurance Inspector, Food and Drug Administration Inspector).

## Results

In the present study, 8 main categories and 26 subcategories were identified using directed content analysis to determine the challenges of drug supply. Therefore, the patient must decide independently (Table 2).

**Table 2:** Challenges of drug supply at the pharmacy level

Principle	Category	Sub-category
**Autonomy**	Patient independence	Participating patient in decision-making Giving patient the required information
	Communication principles	Pharmacist-patient relationship/communication Physician-pharmacy relationship/communication
	Patient privacy	Preserving patient physical privacy Privacy and confidentiality Gender proportion or same-sex care delivery
**Non-maleficence**	Patient harm avoidance	Educational system Pharmacists’ awareness of own professional commitment Not letting pharmacy license Lack of ignorance and not rushing to deliver drug to patient
	Supervision	Pharmacist’s monitoring of drug storage condition in the pharmacy Monitoring the import of foreign and smuggled drugs Advertising drugs based on their effectiveness and not just paying attention to their brand Disposal of pharmacological waste
**Beneficence**	Patient-centered services	Considering patient’s interests Increasing patient’s awareness Sense of responsibility in pharmacological system Acceptable quality of medication
	Drug supplier	Standardization of pharmacies in terms of space, temperature, humidity, and shelving The criteria for entry or exit of drug to drug list Culturizing prescription and rational drug use Drug hoarding by distribution companies
**Justice**	Equitable distribution	Equitable distribution of drugs Justice in the supply and distribution of medicines Equitable distribution of responsibilities and interpersonal interests
	Procedural justice	Justice in law enforcement
	Interactive justice	Justice in protecting people’s rights

### Principle 1: autonomy

Human dignity should be respected in any circumstances, and patients or healthy persons as human beings choose the path of treatment and recovery consciously and autonomously, therefore, information is needed to make decisions. This flow of information takes place in the context of communication principles, and protecting the patients’ privacy is effective in making this connection, and ultimately, making decisions. Therefore, according to the participants’ opinions, for the concept of autonomy, three sub-categories including patient independence, communication principles, and patients’ privacy were identified as follows.

#### Category 1: patient independence

It is the ability to make informed or rational decisions and to act upon them, which are only made in caring situations. In this case, participants emphasized on the patients’ participation in decision-making and giving patients the required information.

Patients participation in decision-making is one of the codes of the patient independence category. A participant in this regard stated that:

“…*Lack of patient participations in decision-making on treatment is a moral challenge and based on the principles of evidence-based medicine, patients must be given the right to choose and it is wrong to use clinical power when prescribing a treatment*…” (Pharmacist 5).

“…*Lack of ethical decision-making models in pharmacies has caused them to not adhere to ethical principles and give, for example, calculus tablet to the patient instead of prescribed calculus syrup regardless of patients’ preferences..*.” (Insurance inspector 1).

Giving the patient the required information for decision-making was another code of patient independence category. The participants in this regard stated that:

“…*The presence of a pharmacist in pharmacies gives patients the right to receive information about drug treatment. Also, all information on how to use drugs in treatment process or other information should be given to patients according to their level of knowledge, literacy, and health status, so that it would be understandable for them and their next of kin ..*.” (Pharmacist 2).

“…*If pharmacists do not give information to patients on how to use the drug and its side effects, they cannot get a favorable outcome from the treatment, so it is useless and dangerous to administer drug without providing information about its use. This is why pharmacists should provide pharmaceutical information as the last treatment loop..*. “(Food and drug administration inspector 4).

#### Category 2: communication principles

Proper communication is a win-win situation for pharmacists, patients, physicians, and generally, for the healthcare system. This communication should be a two-way communication with its own rules. According to the participants’ opinions, this category had two subcategories as follow:

Pharmacist-patient relationship/communication: The majority of participants believed that:

“…*The treatment environment should be so that patients realize staff are there because they are so valuable to them. Also, appropriate relationship and preserving patient dignity regardless of race, skin color, nationality, and income are fundamental to have an effective communication in clinical environment and its absence is a moral challenge..*. “.

“…*Sometimes pharmacists are faced with unethical and unprofessional request, for example, people want cosmetic drugs instead of prescription drugs, or want illegal documents for insurance without a prescription. Such issues are due to the unethical relationship between patients and pharmacists*...” (Pharmacist 4).

“…*The relationship between pharmacist and patient is a relationship between two people that should be based on respect and trust, but unfortunately, for a number of reasons, including self-interest, communication principles are not respected properly*…” (Pharmacist 5).

Physician-pharmacy relationship/communication: Treatment process makes physicians to have a close relationship with pharmacists, but sometimes, the physician-pharmacy relationship presents an ethical challenge. Participants in this regard stated that:

“…*The family relationship between physicians and pharmacists is one of the ethical challenges in this field, and physicians should prescribe medication based on patients’ needs, but sometimes, it is based on the financial interests and other motives. For instance: the daughter of a pharmacy owner is a dermatologist who send her clients to her father’s pharmacy, justifying it by saying that, this pharmacy reads the prescription better, it is more skillful in making mixed medicines, and it has a complete list of drugs without a deficit*. “ (Insurance Inspector 2).

“…*Occasionally, there is a contract between physicians and pharmacists in the way that, pharmacists give a list of its near-term medications and physicians prescribe these unrelated drugs to patients*….” (Patient 5).

#### Category 3: patient privacy

According to the participants’ perspectives, one of the things that should be considered in autonomy and is a context for two subcategories of autonomy in decision making and having effective communication, is protecting patient’s privacy, which refers to protecting the physical space privacy, confidentiality, and information privacy, as well as gender proportion.

Preserving patient physical privacy is one of the codes found in this study. In this regard, some participants stated that:

“…*Physical privacy is a place that surrounds a person’s body and is actually a protected area for the person. The pharmacies’ space should be so that it creates an opportunity for respecting people’s privacy*…” (Patient 4).

“…*A pharmacy is an environment where patients have fewer facilities and more often their privacy is not respected and this is a moral challenge which inflicts harm*…” (Pharmacist 2).

“…*Patients’ privacy is not respected in pharmacies due to the lack of physical space. Physical privacy means that there must be some private space between them and other clients when buying drugs. One of our challenges in the crowded pharmacies is physical contact between male and female clients*…” (Pharmacist 2).

Privacy and confidentiality is another code related to the patient privacy. Participants in this regard stated:

“…*This issue is not specific to Iran, and countries such as the United States, Britain, and Scotland also insist that pharmacists and pharmaceutical technicians should strive to respect patients and protect their right to confidentiality..*.” (Pharmacist 1).

“…*Patients wish their pharmaceutical information to be kept confidential by pharmacists. The concern of some people when visiting a pharmacy is that their colleagues, friends, and neighbors will see or hear their pharmaceutical information..*.” (Patient 4).

“…*This is one of the most important ethical issues in the pharmacy environment, which indicates the need for serious and practical commitment of the pharmacy’s staff. They are also obliged to adhere to this right by Sharia and law. But a problem that I have always seen in Kerman province is that patients’ privacy and confidentiality is not important to pharmacists and pharmacy staff..*.” (Insurance inspector 1).

Gender proportion or same-sex care delivery: Participants believed that gender proportion is effective in the relationship between patients and pharmacists, in maintaining the patients’ privacy, and reducing stress, and subsequently, in making patients autonomous, so that one of the participants stated:

“…*People tend to get their medication and ask their questions from their same-sex peers, and adhering to the principles of same-sex care in the health sector will increase the patients’ morale and reduce their stress when receiving a service..*.” (Pharmacist 7).

### Principle 2: non-maleficence

In any therapeutic action, it is a key principle to not inflict harm or injury on patients, and the pharmacy must take steps that their benefits outweigh the risks. According to the participants’ opinions, avoiding harm to the patient, monitoring the conditions of pharmacies environment, and management of the pharmaceutical warehouse and its prescription by the providers were identified, which is discussed below.

#### Category 1: patient harm avoidance

Participants also pointed out that in order to avoid harm to the patients, the education and learning system plays an important role in observing ethical principles in this regard, and pharmacists must be aware of their professional obligations and leave their pharmacy license to someone else and do not hurry and neglect the process of prescribing and delivering medicines to the patients.

Educational system: It has an important role in teaching the principles of pharmaceutical ethics, so that one of the participants stated that:

“…*There is no related module/course for pharmacy students. After graduation, pharmacists start working in pharmacies while they are unaware of the principles of pharmaceutical ethics and the rules related to their pharmaceutical activities. They think that pharmacy like local supermarkets is a place for selling drugs. This issue can cause irreparable damage to the country’s healthcare system...*” (Pharmacist 5).

Pharmacists’ awareness of own professional commitment is one of the codes of this category. One of the participants in this regard stated that:

“…*The pharmacist must be aware of the requirements and principles within the pharmacy system and prevent any harm to patients and pharmacies. Professional tasks should be done by pharmacists, and pharmacy technician should not be allowed to arbitrarily and unknowingly prescribe medication, and subsequently, affect the health system..*.” (Inspectorate of food and drug administration 1).

Not lending pharmacy license: A participant believed that:

“…*Lending pharmacy license is one of the ethical challenges in the pharmacy system in our country. Pharmacy graduates in the country lend their pharmacy license to investors at exorbitant prices. Pharmacists, who do not have 10 billion Rials to buy a property and medicines to establish a pharmacy, lend their license to an investor. Is it a pharmacy or a supermarket? The main issue is avoiding harm to patients.*” (Pharmacist 3).

Not neglecting or rushing to deliver drug to patients are effective measures to prevent harm to patients. One of the participant in this regard stated that:

“…*Pharmacists should manage their pharmacy by giving priority to patients and avoiding patients’ harm...*” (Pharmaceutical Technician 3).

*“*…*In a pharmacy, the manager rated technicians based on the number of prescriptions they prepared, which increased their carelessness and negligence. Patients’ life depends on medication and negligence puts patient’s life in danger, which its moral responsibility is on the pharmaceutical technicians*...” (Deputy Inspector 1).

#### Category 2: supervision

Supervision is one of the subcategories, which according to the participants’ opinions, it can prevent harm to patients. Effective and efficient monitoring and control of drug storage conditions, the drug supply and distribution methods, especially smuggled drugs, and monitoring drug advertising are essential.

Pharmacist’s monitoring of drug storage conditions in the pharmacy: It is one of the important codes related to monitoring. As stated by one of the participants:

“…*Due to the presence of reactive and supplementary substances in medicines, heat, cold, humidity, and light affect the nature of drugs*...” (Pharmacist 1).

“…*In the pharmacy evaluation guideline, which is issued every 3 months, the storage condition has 50 points out of 1000 evaluation points. Although this indicator is very important and affects the quality of drugs, its point is much less than other indicators*…” (Food and drug administration’s inspectorate 1).

Monitoring the import of foreign and smuggled drugs: Insufficient monitoring of the import of foreign drugs may cause irreparable damages to patients. In this regard, one of the participants stated that:

“…*Currently, the import of foreign complementary medicines into the country has increased, and while the country is facing the shortage of resources to meet drug needs, cost-effectiveness research should be conducted regarding the import of complementary drugs*…” (Pharmacist 6).

“…*A large number of drugs is currently imported and some of these drugs have national samples. Restrictions of pharmaceutical products import have created many challenges in the pharmaceutical field that need supervision*…” (Pharmacist 2).

“…*Iran’s sanctions have led to the smuggling of a large volume of drugs in the country, and the types of supervision is one of the causes of this problem...*” (Patient 4).

Advertising drugs based on their effectiveness and not just paying attention to their brand: According to the participants’ statements, one of the current challenges in pharmacies is supplying drug based on their brand but not their effectiveness. In this regard, a participant stated that:

“*…Many physicians, including dermatologists, prescribe branded medications advertised by drug salesmen without knowing their safety and effects on health...*” (Insurance inspector 2).

“*…Prescribing an effective drug ensures positive treatment outcome and now prescribing foreign drugs are being prohibited by the internal physicians under Section 6 of the Developmental Law. This is while the quality of national drugs is not the same as foreign ones and this paves the way for drug trafficking…*” (Pharmacist 3).

Disposal of pharmaceutical waste, monitoring the process and doing it well are the issues that should be considered in this regard so that one of the participants stated that:

“…*Disposal of pharmaceutical waste is not done properly, and disposal of drugs or household waste in nature can cause many problems, including microbial resistance*…” (Pharmacist 5).

### Principle 3: beneficent

The health system strives to provide the highest levels of profitability and benefits for the society in line with public health goals. Profitability means doing things that benefit patients. According to the participants’ opinions, the services should be patient-centered and the use of drugs should be optimal and rational, so the following two subcategories related to profitability were identified.

#### Category 1: patient-centered services

Participants also stated that in providing services, patients’ interests, informing patients about how to use drugs and their side effects, the sense of responsibility in the drug management cycle and also ensuring the quality of the drug should be considered; in other words, providing services should be patient-centered. According to the opinions, the following codes were extracted.

Considering the patient’s interest: It is one of the factors highlighted in this study. The participants believed that services provided to patients should be such that, the patients’ benefits override the financial and economic benefits of the pharmacy.

“…*When drug supply in the pharmacy is seen as a business, the patients’ interests go away and the profits from the sale take priority” (Pharmacist 4).*

Another interviewee stated that: *“In pharmacy, financial gain is more important than serving patients, and when the basis is just money, patients will not be in priority. In such a situation, the public call pharmacists as thieves...*” (Food and drug administration inspector 2).

Increasing patient awareness: This code creates psychological and emotional readiness in people facing with anxiety caused by disease. Based on the interviewees’ statements about informing patients, the following cases should be considered:

“…*The patients have the right to know how to take medications. Not giving information or giving wrong information to patients breaks the treatment cycle. In such a situation, patients will not be treated and the treatment will not be effective. However, despite the importance of increasing patients’ awareness both legally and morally, this is not implemented for the sake of pharmacist benefits...*” (Pharmacist 7).

*“…Delivering similar drugs to patients without informing physician and patients is a moral challenge. Pharmacies have the right to consult, but have no right to change the prescription. For example, when a physician prescribed Madopar, pharmacies have no right to replace it with Levodopa..*.” (Insurance inspector 1).

“…*We should inform patients about the near-expiry drugs on the prescription. Patients could be an illiterate old man or woman who keeps the medications for few months without knowing that they will be expired soon..*.” (Pharmaceutical technician 4).

Another interviewee in this regard stated that: “*Patients should be aware of any mistake in insurance deductions created by physicians that could cause them to pay the full charge of drugs.*” (Patient 3).

According to the participants’ comments, *“Information on the technical fees, drug interactions, medication costs, adverse effects of the drug, and how to use the drug are among the issues that patients should be aware of ...”*

Sense of responsibility in pharmaceutical system: It is another issue that was emerged in relation to patient-centered services. In this regard, the participants stated that:

“…*Morality means a sense of responsibility that should be not only in the pharmacists but also in the pharmacy technicians. The technician should check the validity of prescription so that the pharmacy will not be fined..*.” (Pharmacist 6).

“*Pharmacists must give clear and legible information to patients and this responsibility cannot be delegated to pharmacy technicians as this is the last thing that patients do before leaving pharmacies*.” (Insurance inspector 2).

“…*Our challenge is the targeted sources of information, such as visitors of pharmaceutical companies, especially pharma-cosmetic companies that promote expensive, and in some cases, less-effective drugs for more profits. In such situations, nothing but a sense of responsibility and conscience of technical officer can prevent this problem..*.” (Insurance inspector 3).

Acceptable quality of medication is another code related to patient-centered services. In this regard, the participants believed that:

“…*The production of poor-quality drugs is a waste of resources, and sometimes, a risk to people’s health. The production of poor-quality drugs is a problem..*.” (Pharmacist 5).

“…*The herbal medicines that are being distributed and the ointments and creams that are manufactured manually in the pharmacy may not have the required quality. For instance, the X herbal company changes the expiry dates of its products and sends them to pharmacies to be sold to patients. Ointments and creams are also occasionally seen to be made by non-pharmacists..*.” (Pharmacist 8).

#### Category 2: drug supplier

Optimizing drug supply environment is influenced by the following factors:

Standardization of pharmacies in terms of space, temperature, humidity, and shelving: According to the opinions of most participants, this is important as it affects people’s health, but it is less important in the pharmacy environment.

“…*In pharmacies, the condition of drug storage, cleanliness of the pharmacy environment, and the instructions of drug storage in order to achieve the optimal conditions should be monitored and supervised..*.” (Insurance inspector 1).

“…*The physical standards of pharmaceutical care have been developed by the Food and Drug Administration in 8 articles and have been issued to pharmacies and drugstores. This regulation obliged pharmacies to comply with standards regarding temperature, humidity, light, and shelving, but it is not respected and it is not given more importance for many reasons..*.” (Food and drug administration inspector 4).

“…*Sometimes pharmacies use lasers and lighting effects to make cosmetics products more luminous and noticeable, which can affect the chemical formulation of the products and medications and even make them ineffective...*” (Pharmaceutical technician 5).

“…*One ethical challenge that seems to be regular for pharmacy technicians is the use of cooler or heater for air conditioning. At one of my pharmacy inspections, I saw a KPTVA spray that was placed next to the heater with very high temperature and no one was concerned that the heat could decrease its effectiveness. Not controlling the temperature and humidity of pharmacies may cause financial loss and health risks...*” (Food and drug administration inspector 4).

The criteria for entry or exit of drug to drug list is another code related to the optimization of drug supply, which has a significant effect on the patient’s financial interests because insurance companies cover drugs listed on the drug list. In this regard, the participants stated that:

“…*There are currently many drugs on the national drug list that cannot be prescribed at all. When the selection of drugs on the list is based on the proper and evidence-based criteria, the health indicators will also improve..*.” (Pharmacist 4).

“…*Adding or removing drugs from the drug list and updating the drug list is in line with the Sixth Developmental Plan, but since the workgroup is novice, the criteria for drug entry into the drug list is not complete, which causes discontent and challenges among people and pharmacies*…” (Pharmacist 5).

Culturizing prescription and rational drug use: Proper prescription and rational use of medicines is currently one of the most important factors in health care. The resources are seriously wasted in the field of medication. All participants, while emphasizing on the culturizing prescription and rational drug use, believed that:

“…*Culturizing is a very important factor in prescribing and taking medication. If pharmacies send patients home with a large bag of drugs, the patient thinks that the physician is good and this is one of the causes of irrational prescription of drugs...”* (Pharmacist 1).

“…*Physicians know that if they write a simple prescription for patients, they will not get a good response from the patients, and sometimes, patients will not follow-up their treatment. It can also be a bad advertising for the physicians. We have a cultural problem and the prescription and rational drug use is a problem in Iran..*.” (Pharmacist 6).

“…*The factor that causes irrational drug use is deficiency in the faulty system of drug prescription and distribution, which is caused by false propaganda and claims of some pharmaceutical companies and marketers that encourage physicians and pharmacists to use their products..*.” (Insurance inspector 2).

Drug hoarding by distribution companies: Drug hoarding or stoking and refusal to supply drug was another code related to the optimization of drug supply from the participants’ point of view. For example, participants stated that:

“…*The country has been sanctioned by some countries because of political issues. As a result, raw materials and technology are difficult to be imported into the country and this creates a suitable situation for drug trafficking and increasing price. The government must set policies to prevent hoarding…*” (Insurance inspector 2).

“…*Hoarding medicines that are directly linked to health and well-being may endanger the lives of thousands of innocent people, and the primary mission of pharmacists is to benefit society..*.” (Patient 5).

“…*One of the factors that disrupt the optimum drug cycle is that, pharmacists and distribution companies stoke drugs in stores other than their pharmacies’ store due to the shortage of medicines and become drug hoarders...”* (Pharmacist 1).

### Principle 4: justice

The complexity of the issue of justice in the field of medical ethics and the health system is due to its great variety based on philosophy and different evaluation of the concept of ethics. Justice, fairness, and equality should be considered by the participants in issues related to the distribution of scarce health care resources, the process and procedure of decision making about who receives what treatment and how power interactions should be in different positions. Therefore, the codes extracted in this context, were categorized into three subcategories of distributive, procedural, and interactional justice.

#### Category 1: equitable distribution

Participants stated that pharmacy licensing, capital distribution, as well as medicine distribution among pharmacies, labor division, and distribution of roles and responsibilities among individuals should be fair, so that the drug distribution system be equitable.

Equitable distribution of drugs: It was one of the codes highlighted in the present study. The participants believed that:

“…*The transfer of pharmacies’ capital is not fair and it is determined by the brokers according to the insurance-related license and the number of pharmacy sales, which is licensed by the Food and Drug Administration...*” (Food and Drug Administration inspector 3).

Justice in the supply and distribution of medicines: It is another code that was considered in distributive justice and the participants stated that:

“…*There is no justice in the distribution of medicines. Currently, there is the lack of some medicines in Kerman, and larger cities are always in preference for distribution of medicines..*.” (Patient 4).

“*…Quota medicines are not distributed fairly, and we cannot track how many quota medications have been given to each pharmacy. Because top officials in the Food and Drug Administration have their own pharmacy, so it is the best interest of medicine distribution companies to make these powerful people happy….*” (Pharmacist 3).

Equitable distribution of responsibilities and interpersonal interests: It is one of the subcategories of equitable distribution that was highlighted in the present study. In this regard, the participants stated that:

“…*The distribution of responsibilities and interests in pharmacies must be based on the fairness and benefits of patients. Effective communication should also be established between individuals, and this will happen when the tasks are allocated correctly*….”

#### Category 2: procedural justice

Procedural justice is the extent to which the rules and procedures specified by the policies are followed uniformly in all cases. One of the codes extracted from the interviews is justice in law enforcement.

Justice in law enforcement: Seeing individuals equal in law enforcement can provide a good basis for achieving justice in the community. It also increases patients’ trust in the pharmaceutical system. According to the interviewees’ opinions:

“…*People want the law and processes to be implemented equally for everyone. If there is no justice in law enforcement, mistrust is created. Mistrust is the most serious problem in our health system, and if it is the result of injustice in law enforcement, it cannot be corrected by guidelines and regulations*”*.*

#### Category 3: interactive justice

Interactive justice is one of the main categories of justice. People are sensitive to the quality of their interpersonal interactions and tend to have relationships based on politeness, honesty, and respect, especially between patients and healthcare providers. Interactive justice is about the ways by which customers, who received an imperfect service, are treated.

Justice in protecting people’s rights: It is one of the subcategories of justice, which was highlighted in the present study. In this regard, the participants stated that:

“*… Interactive justice is felt when people’s rights are being respected and unsatisfied customer is treated honestly, compassionately, and politely. Pharmacies should treat patients as they would like to be treated by other pharmacies…*”.

## Discussion

In the present study, the challenges of ethical behaviors in the area of drug supply in pharmacies were investigated using a principle-based approach proposed by Beauchamp and Childress. According to the results of a study by Cooper RJ et al. (2007), many empirical ethics research have been conducted in the field of health care, but ethical challenges in pharmacies have not been addressed [12]. This qualitative study investigated ethical behaviors based on the four principles of bioethics and eliminated the suggestion made by Cooper RJ et al. These four ethical principles are measured and judged in interaction with one another, and attracted our attention to the extent and scope of their application, which is discussed below. First, the principle of autonomy gives patients the right to choose or refuse their treatment. Second, the principle of beneficence refers to the fact that healthcare providers must act in the patients’ best interests. The principle of non-maleficence emphasizes that patient should not be harmed. Finally, in the principle of justice, the distribution of healthcare resource and making decisions on who receives what treatment and medication should be considered.

### Autonomy: patient independence

The principle of autonomy determines the rights of individuals in decision-making. This principle refers to giving respect to each individual in the community and the ability to make informed decisions on their own issues. The autonomy principle is discussed in three main categories: patient independence, patient privacy, and compliance with the principles of communication, most of which are related to patient’s autonomy and privacy.

In the study of Lemonidou et al. (2003), patients’ independence was defined in two areas: providing sufficient information to patients and allowing them to make therapeutic decisions [23]. In this regard, the results of other studies reveal that healthcare providers do not respect patients’ autonomy by not involving them in the decision-making process and not providing them sufficient information [24, 25]. In this regard, the Comprehensive Document of Pharmaceutical System and the Patient Rights Charter in Iran and the Patient Rights Charter of the American Hospitals Association [26] emphasize that comprehensible information must be given to patients and they must be involved in decision-making about any treatment or diagnostic process [18, 27]. Another study by Florin et al. (2006) showed that nurses do not involve patients in clinical decision making, while patient participation is very valuable in decision making and improving the quality of services [28]. The present study showed that, this issue is one of the ethical challenges in the field of medicine.

### Autonomy: patient’s privacy

Studies have shown that due to crowded pharmacies and insufficient space in pharmacies, patient’s privacy is not preserved in pharmacies [19]. Wirth et al. (2010) reported that the lowest satisfaction level was related to patient’s privacy in pharmacies [29]. Another health-related study emphasized on the patients’ privacy and confidentiality [30]. Sommerville et al. (2012) also showed that the layout of pharmacy space is such that in about 50% of cases, patients’ physical privacy is not respected [31]. The Patients’ Rights Charter also emphasizes the confidentiality and respect to patients’ privacy [27]. According to the Codes of Ethics proposed by the American Pharmacy Association, pharmacists must strive to promote and protect patients’ privacy [32]. People tend to get their medications and ask their questions from same-sex counterparts, while the UK Patients’ Rights Bill states that women should always have access to a female staff and people who perform examination and counseling must be the same-sex staff [33, 34]. In the present qualitative study, patients’ privacy was investigated and it was revealed that non-compliance with this principle is one of the major challenges in pharmacies.

### Autonomy: communication principles

The review of published studies on the pharmacist-patient communication showed that pharmacist-patient communication is one-way and there is no interactive and collaborative process between them [35–38]. The results of other studies on the pharmacist-patient relationship have shown that the requests that are contrary to professional and ethical responsibility and demands of jobbers can harm ethical communication [39]. Failure to adhere to the principles of communication and non-presence of pharmacists in pharmacies is one of the factors leading to ineffective communication that endangers patients’ interests and undermines community’s trust [40–42]. Studies in other areas, such as physician-pharmacy relationship, have shown that pharmacists’ family relations with physicians, pharmacists’ financial relationship with physicians [31], and self-referral are among ethical challenges of physician-pharmacist relationship that reduces people’s trust and confidence, increases health care costs, and leads to the commercialization of treatment and medication in patients’ mind, which in turn, promotes unethical relationship between physician and pharmacist [43]. In the United States, to combat professional misconduct between pharmacists and physicians, the Stark law was established, according to which, physicians do not have the right to send patients to places that benefit them [44]. By examining this principle qualitatively, the present study showed that non-compliance with communication principles is one of the major challenges in pharmacies. In this regard, a conflict of interest among different roles should be concerned.

### Non-maleficence: patient harm avoidance

In this principle, the most important issue for healthcare staff is to not harm patients and do their best with proper supervision. This is partly due to the fact that pharmacists believe they are doing well, without properly evaluating their performance and making sure that it does not harm patients. The principle of non-maleficence will be discussed in two main categories, including patient harm avoidance and supervision.

Studies have shown that the weakness of educational system in teaching the principles of pharmaceutical ethics is one of the causes of harm to patients [32]. Studies in other areas, such as teaching ethics to medical students, have shown that topics of medical ethics have been sporadically included in the pharmacy curriculum and many of the professional misconducts are due to the weakness of educational system in teaching ethics [45–48].

Lack of pharmacists awareness of their professional obligations is one of the ethical challenges in this field. Studies have shown that pharmacists are professional people who should be aware of their professional obligations and know and adhere to drug and pharmacy standards, however, pharmacists’ lack of knowledge about social and behavioral sciences, as well as ethical concepts and values creates ethical challenges [9, 49].

### Non-maleficence: supervision

One of the most important principles of professional ethics is the principle of non-maleficence, which is closely related to the concept of supervision in pharmacies. The results of studies, while emphasizing on the monitoring of drug storage in pharmacies, show that not only drug storage condition in pharmacies is not supervised, but also there is no supervision in other areas of the health system, including diagnosis, prescription, distribution, and drug use, and this issue imposes a heavy cost on the health system [50].

Studies have shown that lack of supervision over drug advertising causes financial gain to be replaced with effectiveness and quality, and also prescribing brand medication to receive financial reward damages the professional responsibility of physicians and pharmacists [39]. A study by Hosseini (2009) on upholding consumer rights in advertising and marketing of pharmaceutical products showed that articles related to pharmaceutical advertisements are vague, outdated, and irrespective of world-wide developments and consumers’ rights [51]. The present study showed that lack of supervision over pharmaceutical advertisements is one of the major challenges in the field of drug supply in pharmacies.

### Beneficence: patient-centered services

Beneficence refers to measures that improve the well-being of others. It also refers to activities that benefit patients, however, there is no certainty. Based on the findings of the present study, beneficence has two main categories, including provision of patient-centered services and optimization of drug use. This category is also one of the most important ethical challenges in pharmacy.

The results of a study showed that providing services that take into account the patient’s interests and not misleading patients are among ethical principles in the pharmaceutical profession, and pharmacists should prefer the consumer’s interests over their own economic interests, and if they ignore patient’s interests and the principle of beneficence, no service can ethically satisfy the consumers [27, 52, 53]. A study in Australia on the professional ethics in pharmacies showed that pharmacists had many ethical issues and patients’ best interests were considered as the main framework that they must act upon. They also considered financial problems as the most important factor that causes young pharmacists to ignore patients’ interests when making decisions based on the professional ethics [54]. Studies in other areas, such as dentistry, show that prioritizing personal interests and financial gain over duties that they are expected to do as professionals contradicts ethical principles [52]. In this regard, studies of Patient’s Rights Charter and code of ethics in the National Comprehensive Document of Pharmaceutical System have shown that pharmacists must accept the responsibility as the guardians of health care quality and should only provide medications that have acceptable quality [27]. The present study showed that failure to provide patient-centered services is one of the main challenges in the field of drug supply.

### Beneficence: optimization of drug use

A study aimed at prioritizing the factors affecting the quality of pharmacy services showed that standard conditions of pharmacies in terms of space, temperature, humidity, and shelving and overall suitability of physical environment lead to satisfaction of costumers and beneficence [51]. Also, international studies on the quality of pharmacy services reveal that tangible and physical factors such as standards of the pharmacy and suitability of physical environment affect the quality of pharmacy services and benefit both patient and pharmacy [24, 26, 49, 55]. These studies suggest that, pharmacies should be standardized in terms of space, temperature, humidity, and shelving, which is consistent with the results of the present qualitative study.

### Justice: distributive justice

Justice means putting everything in its proper place, which is institutionalized within the human being and encompasses all individuals and social areas of life. The principle of justice as one of the four principles of the professional ethics was discussed in the present study in three main categories, among which distributive justice was the biggest ethical challenge in the principle of justice.

The results of studies have emphasized the importance of justice in drug supply and distribution [39]. According to the results of the present study, drug shortage affects equity in drug supply to patients. Failure to adhere to justice in the distribution of resources undermines the professional responsibility of pharmacists, and as a result, causes mistrust among patients. However, Article 4 of the Comprehensive Document on ethics of Pharmacy System states that pharmacists must be fair in the distribution of health resources and they should be aware of any issues that undermine their ethical responsibilities. The Charter of Patients’ Rights in Iran emphasizes the equitable distribution of healthcare resources and the prevention of waste of resources [50].

### Justice: procedural justice and interactional justice

In this study, participants reported procedural and distributive justice as ethical challenges of justice. Article 4 of the Code of Professional Ethics states that pharmacists must contribute to the promotion of community health fairly and equitably [18]. A study investigated the outcomes of ethical values in an organization showed that ethical values affect procedural and interactional justice. When employees perceive fairness in the procedures, they strive to undertake ethical behaviors [54]. These studies show that pharmacies should regulate their ethical behaviors based on justice, which is consistent with the results of present study.

### Study limitations and strengths

In this study, a common principlism approach was used as a guide to explain what can ethically occur in behavers of practitioners in drug supply in an area of Iran which is extremely important in the development of a qualitative research and makes the results more insightful. This study also had some limitations. One of the limitations is that some stakeholders were reluctant to participate and engage in the study as the interviewer tried to minimize it through emailing an information sheet about the detailed description of study and providing enough opportunity to participate in the study. Another limitation is that the interviews were limited to the most formal stakeholders in a local setting, and it did not include other formal and informal actors such as drug dealers in informal markets, drug manufacturers, drug importers, and drug distribution companies in a wider national context. Therefore, some ethical challenges in the supply chain in Iran, can be potentially neglected. Thus, the results of this study are not inclusive and generalizable particularly for other areas in Iran or other healthcare systems around the world as this is a qualitative study and generalizability is not the subject of these studies [56]. Alternatively, we pursued transferability by presenting information that others need to determine the relevance of the results to their particular contexts. And the last limitation is the reliance on interviews as a single source for data collection, which requires further research to be conducted with triangulation of data collection methods.

## Conclusions

In the present study, the challenges of ethical behaviors in the supply of medications in pharmacies based on a principle-based approach were investigated. The results showed that most ethical challenges in drug supply were related to the aspect of autonomy with subscales of patient’s independence and privacy, and aspect of beneficence with subscale of patient-centered services. Regarding the subscale of patient-centered services, attention should also be paid to increasing patient awareness, culturizing prescription and rational drug use, confidentiality and privacy, and pharmacist-patient communication. This study can be used as a tool for introducing ethical challenges to policymakers. It can also help create a moral environment by reviewing drug supply policies. The Ministry of Health and other health and education authorities are suggested to dedicate ethical modules and seminars to the pharmacy curriculum to reduce the ethical challenges of drug supply. Patients should be involved in making decisions about their own issues and should also make decisions based on the perceived information, accordingly, the patient’s independence and ethical commitment are respected. Institutionalizing ethics and ethical responsibility when prescribing medications based on the effectiveness creates trust and increases productivity. Pharmacies environment should also be designed in such a way that facilitate patient communication with pharmacists and protect patients’ privacy.

Using the results of the present study, the Food and Drug Administration can develop an ethics charter and inform pharmacists and pharmacy technicians about this code. It can also use measures like compliance with ethical codes in granting and extending pharmacy licenses.

Given that many approaches and theories have been presented in the field of ethical behavior and there is no ethical theory covering all considerations of experts in the field of work ethics, therefore, it is suggested to evaluate other ethical approaches.

## Supplementary information

**Additional file 1.** Interview guide

## Data Availability

The transcribed interviews and open coding are available from the corresponding author on a reasonable request. It should be noted that all interviews were conducted, transcribed, and are accessible in the Persian language.

## Abbreviations

MI: Mahla Iranmanesh
VYF: Vahid Yazdi-Feyzabadi
MHM: Mohammad Hossein Mehrolhassani

## References

[1] Furber C (2010). Framework analysis: a method for analyzing qualitative data. Afr J Midwifery Womens Health.

[2] Gallagher CT (2011). Assessment of levels of moral reasoning in pharmacy students at different stages of the undergraduate curriculum. Int J Pharm Pract.

[3] Kiani M (1390). Medical Ethics and Law. Iran J Med Sci Org.

[4] Buerki RA, Vottero LD (2002). Ethical responsibility in pharmacy practice: Amer. Inst. History of Pharmacy.

[5] EbadifardAzar F, Rezapoor A, Rahbar A, Hosseini Shokouh SM, Bagheri FS (2013). Estimation of the function of medicine demand in Islamic Republic of Iran. J Mil Med.

[6] Joodaki H, Rashidian A (2010). Review of corruption in the health sector: theory, methods and interventions. JHOSP.

[7] Farsam H (2008). The pathology of pharmacy ethics. IJME.

[8] Fazeli Z, Fazeli Bavand Pour F, Rezaee Tavirani M, Mozafari M, Haidari Moghadam R. Professional ethics and its role in the medicin. SJIMU. 2013; 20 (5) :10–17.

[9] Wingfield J, Bissell P, Sadler S (2003). Student perceptions of pharmacy ethics. Int J Pharm Pract.

[10] Masoumeh Sadat Saeedi, Alireza Faghihi, Mohammad Seifi, Faezeh Nateghi. Investigating the position of professional ethics and presenting a favorable pattern of its development. Q J Educ Leadersh Adm., 12(2), 107–124.

[11] Nikkhah Farkhani Z, Khorakian A, Jahangir M, Shahrodi HM (2018). Explaining the Components of Ethical Behavior in Employees & Managers of the City. J Ethics Sci Technol.

[12] Cooper R, Bissell P, Wingfield J (2007). A new prescription for empirical ethics research in pharmacy: a critical review of the literature. J Med Ethics.

[13] Ross WD (2012). What makes right acts right?. Ethics.

[14] Al-Arifi MN (2014). Community pharmacist perception and attitude toward ethical issues at community pharmacy setting in Central Saudi Arabia. Saudi Pharm J.

[15] Kruijtbosch M, Göttgens-Jansen W, Floor-Schreudering A, van Leeuwen E, Bouvy ML (2018). Moral dilemmas of community pharmacists: a narrative study. Int J Clin Pharm.

[16] Salari P, Abdollahi M (2017). Ethics in pharmacy curriculum for undergraduate pharmacy students: a needs assessment study. Arch Iran Med.

[17] Lowenthal W (1986). Klein 3rd WS, Overton CP. Thinking about ethical dilemmas in pharmacy. Am J Pharm Educ.

[18] Comprehensive Ethical Document of the Pharmaceutical System of the Country.Medical Ethics and Law Research center (MELRC).Shahid Beheshti University of Medical Sciences.

[19] Reisnejadian S, Ebrahimi S, Hemmati S (2016). Ethical challenges in community pharmacy setting from the perspective of Shiraz School of Pharmacy faculty members and pharmacy practitioners: a qualitative study. IJME.

[20] Lee MJH (2010). The problem of ‘thick in status, thin in content’ in Beauchamp and Childress' principlism. J Med Ethics.

[21] Hsieh H-F, Shannon SE (2005). Three approaches to qualitative content analysis. Qual Health Res.

[22] Guba EG, Lincoln YS (1982). Epistemological and methodological bases of naturalistic inquiry. ECTJ..

[23] Lemonidou C, Merkouris A, Leino-Kilpi H, Välimäki M, Dassen T, Gasull M, Scott PA, Tafas C, Arndt M (2003). A comparison of surgical patients’ and nurses’ perceptions of patients’ autonomy, privacy and informed consent in nursing interventions. Clin Eff Nurs.

[24] Bastani P, Dorosti H, Ahmadzadeh M (2017). A survey on Pharmaceutical System of Shiraz Pharmacies from the Clients` View Points. J Healthc Manag.

[25] Rahmani A, Ghahramanian A, Mohajjalaghdam AR, Allahbakhshian A (2008). Perception of patients regarding respecting to their autonomy during nursing care in hospitals affiliated to Tabriz University of Medical Sciences. Iran J Nurs Res.

[26] Mossadegh Rad AM, Esna AP (2004). Patients and physicians awareness of patients’ rights and its implementation at Beheshti hospital in Isfahan. Iran J Med Educ.

[27] Parsapoor A, Bagheri A, Larijani B (2010). Review of the patient's right charter revolution. IJME.

[28] Florin J, Ehrenberg A, Ehnfors M (2006). Patient participation in clinical decision-making in nursing: a comparative study of nurses’ and patients’ perceptions. J Clin Nurs.

[29] Wirth F, Tabone F, Azzopardi LM, Gauci M, Zarb-Adami M, Serracino-Inglott A (2010). Consumer perception of the community pharmacist and community pharmacy services in Malta. J Pharm Health Serv Res.

[30] Schwartz B (2009). An innovative approach to teaching ethics and professionalism. J Can Dent Assoc.

[31] British Medical Association. Medical ethics today: the BMA’s handbook of ethics and law 2nd edn. London: BMA; 2004. p. 648–9.

[32] Resnik DB, Ranelli PL, Resnik SP (2000). The conflict between ethics and business in community pharmacy: what about patient counseling?. J Bus Ethics.

[33] Woogara J (2005). Patients' rights to privacy and dignity in the NHS. Nurs Stand.

[34] Peymani Z, Sherafat M, Mahmoodiyan F (2009). Evaluation of sex proportion to health care staff in operating room: an ethical evaluation. Iran J Med Ethics Hist Med.

[35] Shah B, Chewning B (2006). Conceptualizing and measuring pharmacist-patient communication: a review of published studies. Res Soc Adm Pharm.

[36] Azhar S, Hassali MA, Ibrahim MI, Ahmad M, Masood I, Shafie AA (2009). The role of pharmacists in developing countries: the current scenario in Pakistan. Hum Resour Health.

[37] Olsson E, Ingman P, Ahmed B, Sporrong SK (2014). Pharmacist–patient communication in Swedish community pharmacies. Res Soc Adm Pharm.

[38] Latif A, Boardman HF, Pollock K (2013). Understanding the patient perspective of the English community pharmacy medicines use review (MUR). Res Soc Adm Pharm.

[39] Esmalipour R, Parsa M (2017). The conflict of interest in pharmacy practice. Iran J Med Ethics Hist Med.

[40] Mostafavi SA, Zolfaghari B, Mazrui M, Ekvan A. The Investigation of the patients' Attitudes towards the Relationship with Pharmacist. J Payees. 1383(3):2.

[41] Bagheri A (2011). Iranian medical ethics priorities: the results of a national study. Iran J Med Ethics Hist Med.

[42] Worley MM, Schommer JC, Brown LM, Hadsall RS, Ranelli PL, Stratton TP, Uden DL (2007). Pharmacists' and patients' roles in the pharmacist-patient relationship: are pharmacists and patients reading from the same relationship script?. Res Soc Adm Pharm.

[43] MacKenzie CR, Cronstein BN (2006). Conflict of interest. HSS J.

[44] Parsa M, Aramesh K, Larijani B. A comparison between conflict of interest in Western and Islamic literatures in the realm of medicine. J Med Ethics Hist Med. 2014;7(7):1-6. PMC4263381 25512828

[45] Kiani M, Abbasi M, Sheikh Azadi A, Safar Cherati A, Bazmi S. Ethical requirements in medical education. J Mediev Hist. 2011; Third Year No 8. [In Persian].

[46] Eckles RE, Meslin EM, Gaffney M, Helft PR (2005). Medical ethics education: where are we? Where should we be going? A review. Acad Med.

[47] Khaghanizadeh M, Maleki H, Abbasi M, Abbaspoor A (2011). Identity of the medical ethics curriculum based on the experiences of teachers of medical ethics: qualitative research. Med Ethics J.

[48] Duquette LM. Effects of nursing education on the formation of professional values. [Thesis]. University of Toronto; 2004.

[49] Ghaffari F (2009). Ethical and scientific approach of the book of Minhadj-al dukkan in pharmacy. Med Ethics J.

[50] Abbasi M, Zamani M, Ganjbakhsh M (2010). Justice in medical ethics. Med Ethics J.

[51] Hosseini M (2009). Consumer rights in advertising and marketing of pharmaceuticals and health products. Iran J Med Ethics History.

[52] Parsa M, Khorshidian A (2017). Conflict of interest in dentistry. Iran J Med Ethics Hist Med.

[53] Imaz M, Eteraf-Oskouei T, Najafi M (2018). Evaluation of pharmacy professional ethics in drugstores and its improvement strategies from the viewpoint of students and faculty members of Tabriz School of Pharmacy. Iran J Med Ethics Hist Med.

[54] Baharifar E, Javaheri-Kamel M (2010). The consequences of ethical values. Police Hum Dev J.

[55] Abdolahi Vishkahi S, Zhian P (2017). An inquiry in physician liability in providing drug information with comparison with common law (learned intermediary rule). Iran J Med Law.

[56] Denzin NK, Lincoln YS, Denzin NK, Lincoln YS (2005). Introduction: the discipline and practice of qualitative research. The sage handbook of qualitative research.

