# Supplementary material for: The challenges of ethical behaviors for drug supply in pharmacies in Iran by a principle-based approach
Source: BMC Med Ethics. 2020 Sep 1;21:84. doi: 10.1186/s12910-020-00531-0 (PMC7466816; doi:10.1186/s12910-020-00531-0)
Supplement: Supplementary file 1 — Additional file 1. Interview guide [file 12910_2020_531_MOESM1_ESM.docx]

**‘Explaining of Unethical Behaviors in Pharmacy Settings in Kerman, an Area Located in Southeastern of Iran: Identification of challenges’**

**Mahla Iranmanesh**

Interview guide

*Date & venue:*

*Position of interviewee:*

1. What is your understanding of the concept of ethics and ethical principles in pharmacy settings?
2. What unethical/immoral behaviors occur in the relationship between the pharmacy and the patient when receiving the prescription and delivering the medicines? (Please describe what happens about the patient's interests and position considering authority, beneficence, non-maleficence, and justice).
   - 1. e.g., patient privacy
     2. e.g., the confidentiality of patient information
     3. e.g., medical benefits and medication advice, etc.
3. In your opinion, what unethical/immoral behaviors occur during the distribution and exchange of drugs (communication/interaction between pharmaceutical dispensing companies and pharmacies)?
4. In your opinion, what immoral behaviors occur in the pharmacy's interaction and communication between the medical services providers (doctors, hospitals, clinics, etc.)?
5. In your opinion, what immoral behaviors occur in the pharmacy's interaction with health insurer organizations (Iran Health Insurance Organization, Social Security Organization, Armed Forces Medical Services Insurance Organization, Imam Khomeini Relief Foundation Health Insurance, etc.)?
6. In your opinion, what unethical/immoral behaviors occur in the pharmacy setting about co-workers and their working interactions?
7. What should be the appropriate social behavior in the pharmacy environment?
8. What is your opinion regarding the involvement of the professional pharmacist in pharmacies and their role in pharmaceutical management?
   - 1. Please discuss the pharmacist working conditions and response to patients and clients
     2. Please discuss the pharmacist working conditions, the communication between staff, and workflow in the pharmacy
     3. Please describe what is happened about accessibility to the technical manager (pharmacist) in working hours of pharmacy
9. Please discuss food and medicine directives and bylaws delivered by health authorities and adherence to them. (Challenges, pitfalls, etc.)
10. In your opinion, what immoral/unethical behaviors occur in the process of medicine management (e.g., medicine circulation in pharmacy, dealing with expired drugs, prescription of medicines, over-the-counter (OTC) medicines?)
11. Why do you think these immoral/unethical behaviors occur in pharmacy settings and interact with different stakeholders? Please discuss your solutions to prevent unethical behaviors.
12. Is there anything else you would like to add to the ethics at the pharmacy?
